# Understanding rhizospheric microbial dynamics in gladiolus corms through quorum sensing and quorum quenching for disease control and growth promotion

**DOI:** 10.1186/s12870-024-05722-0

**Published:** 2024-10-23

**Authors:** Akhtar Hameed, Kashif Riaz, Sahar Jameel, Hafiz Muhammad Usman Aslam, Muhammad Waqar Alam, Muhammad Saqlain Zaheer, Muhammad Waheed Riaz, Muhammad Rizwan, Reem M. Aljowaie, Mohamed S. Elshikh

**Affiliations:** 1 Institute of Plant Protection, MNS- University of Agriculture Multan, Multan, 61000 Pakistan; 2 Production Lareault, 90 rue Lareault, Lavaltrie, Québec J5T 3H3 Canada; 3 University of Narowal, Narowal, 51600 Pakistan; 4 Department of Plant Pathology, San Luis Valley Research Center, Colorado State University, Fort Collins, USA; 5 Department of Plant Pathology, University of Okara, Okara, 56300 Pakistan; 6 Department of Agricultural Engineering, Khwaja Fareed University of Engineering and Information Technology, Rahim Yar Khan, 64200 Pakistan; 7 State Key Laboratory of Wheat Breeding, Group of Wheat Quality and Molecular Breeding, College of Agronomy, Shandong Agricultural University, Tai’an, Shandong 271000 China; 8 Department of Plant Nutrition, Institute of Crop Science and Resource Conservation (INRES), University of Bonn, Bonn, 53115 Germany; 9 Department of Botany and Microbiology, College of Science, King Saud University, P.O. 2455, Riyadh, 11451 Saudi Arabia

**Keywords:** Gladiolus, Rhizospheric bacteria, Characterization, Disease management, Growth enhancement

## Abstract

Gladiolus, a widely cultivated cut flower known for its aesthetically pleasing multicoloured spikes, has earned significant commercial popularity. A comprehensive understanding of the rhizosphere bacterial community associated with gladiolus is imperative for revealing its potential benefits. Molecular characterization is considered an effective method to gain insights into the structural and functional aspects of microbial populations. The soil characteristics and bacterial communities in the rhizosphere are typically influenced by quorum sensing (QS) and quorum quenching (QQ) mechanisms. This study aims to explore the niceties and diversity of rhizospheric bacterial populations linked with gladiolus corms, with a specific focus on understanding the dynamics of QS and QQ mechanisms in their complex interactions. The isolation of bacterial strains was achieved through the serial dilution method on nutrient agar (NA) media. The identification of the isolates was accomplished by amplifying 16 S rRNA gene sequences via polymerase chain reaction (PCR) via the use of universal primers. Sequence analysis was conducted via BLAST on the National Center for Biotechnology Information (NCBI) database. The characteristics of the isolated bacteria were elucidated via biosensors. This study identified three QS strains and five QQ strains. A consortium of quenchers was formulated utilizing five strains that demonstrated efficacy in mitigating the impact of disease on gladiolus and fostering growth. Among the three treatments—Scale, Descale, and Descale and Cut Half (DSC)—the DSC treatment emerged as the most effective. This treatment exhibited a broader range of variation in biological parameters over time, aligning with prevailing trends in the local market.

## Introduction

Floriculture exhibits considerable export potential, positioning itself for swift advancement as a distinct subsector within agriculture, attributable to its substantial demand in the global floral trade. Gladiolus (*Gladiolus grandiflorus* L.) is commonly recognized as a sword lily [1] and is highly important as an ornamental plant. Its utilization predominantly involves commercial applications as a cut flower, with occasional instances of incorporation for landscape purposes. The increasing popularity of Gladiolus is attributed to its extensive spectrum of colors and shapes, coupled with its broad adaptability across diverse geographical regions and commendable vase life [2].

*Gladiolus grandiflorus* falls under the Iridaceae family, which comprises more than 2000 identified species. This plant is grown in different major growing areas of Pakistan and has ample flowering in the spring and summer months [3]. Owing to the increased demand for flowers that are used in bouquets, garlands, and other decorative purposes for weddings and other functions, gladiolus, which produces attractive cut flowers on tall spikes, has also been cultivated in Pakistan [4].

The use of chemical fertilizers has reached its peak, has negatively affected the nutrient balance of the soil and has also reduced the quality of the soil for the growth and development of the spikes, corms and cormels of gladiolus [5]. Consequently, the primary concern is to optimize the utilization of nutrients and discharge with the aim of delivering superior-quality products that are not detrimental to the environment. Soil management practices, such as the use of organic soil amendments, microbial cultures and minimal chemical fertilizers, could be useful for improving the biological and physicochemical properties of soil to increase nutrient uptake efficiency. Microbial culture, as a pivotal component of integrated nutrient application in agriculture, offers a prospective avenue for harnessing microorganisms to optimize crop yield, aligning with the principles of environmentally conscious and sustainable organic agriculture [6].

Organic agriculture, which aims to bolster biological cycles, biodiversity, and soil biological activity, strives to establish an environmentally, socially, and economically viable organic system. The rhizospheric population plays a crucial role in ecological processes such as organic debris decomposition, nutrient cycling, and the promotion of crop growth and health through biofertilization [7]. Understanding the composition of the rhizospheric population specific to gladiolus is key for maximizing the benefits conferred by soil microbial communities. While the exploration of plant‒microbe interactions is fundamental, traditional techniques encounter challenges in culturing approximately 99% of microbes [8]. Plant microorganisms, which are influenced by root exudates, soil type, and rhizosphere microbial populations, play pivotal roles in regulating soil and plant health through quorum sensing (QS) [7, 8].

The concept of quorum sensing is the signal regulation of gene expression with respect to shifts in the cell density population; therefore, it is imperative to identify the numerous microorganisms that are involved in this process. There are different types of QQ mechanisms that can effectively suppress sensing systems; this can be due to the degradation of the signal or even interference with the possibility of signal generation and reception [9]. To elucidate the intricate and versatile nature of bacteria in the rhizosphere, it is crucial to focus on the structural and functional characteristics of these bacterial populations in relation to QS and QQ, uncovering aspects of endogenous functioning in plants. This study aimed to evaluate the intricacy and diversity of cultivable bacterial micro biomes closely associated with gladiolus corms. Emphasis is placed on unraveling the complex interactions facilitated through QS and QQ mechanisms exhibited by these rhizospheric bacterial populations.

## Materials and methods

### Field preparation

The experimental site, encompassing an area of 1982.88 sq. ft, was established with ridges measuring 15–25 cm in height and a corm-to-corm distance of 5–10 cm. Manual sowing of corms was conducted at a depth of 8 cm on the ridges, employing four different methods: scaled, descaled, descaled and cut in half, and a combination of all three factors was used as a control. Irrigation was subsequently applied after seeding. A total of 507 corms were sown via a randomized complete block design (RCBD). The research spanned two years, with the first year focusing on evaluating the impact of diseases on plants, isolating microbes, and conducting molecular characterization of the isolated microbes. In the second year, the isolated microbes were introduced to the field, excluding the control, to assess the germination percentage, disease impact, and flower and corm yield. The QQ consortia were established by introducing five bacteria with quorum quenching capabilities sourced from the gladiolus field. The bacterial mixture was applied during irrigation at the 3rd and 5th leaf stages of the plants. The characteristics of the soil observed before the commencement of this study are presented in Table 1.

**Table 1:** Characteristics of the experimental area soil

Parameters	Percentage
Soil texture	Sandy loam
Sand	48%
Silt	32%
Clay	20%
Organic matter	0.59%
pH	7.02
EC	1.27 dSm^− 1^

### Data collection

Data collection was systematically executed at multiple developmental phases of the plant. Specifically, observations were made at distinct leaf stages, including the 1st, 3rd, 6th, and 9th leaf stages. Metrics such as plant length and leaf count were meticulously recorded. Additionally, a comprehensive examination was conducted to identify and characterize diseased and infected segments of the plant on the basis of visual assessments.

### Isolation and identification of pathogens

For the isolation of bacterial pathogens, nutrient agar (NA) media was used under aseptic conditions. The corms and afflicted plant segments were precisely sectioned into 5 mm fragments, ensuring the inclusion of healthy portions. These sections subsequently underwent surface sterilization via distilled water, followed by 0.1% HgCl_2_ treatment and a final rinse with distilled water, followed by thorough drying. The treated samples were then placed onto prepared NA plates, securely wrapped with tape, and incubated at 25 °C for a period of 24 h. Colonies that manifested were subjected to purification via the streaking method. All the purified isolates underwent morphological identification, involving the observation of color, colony type, and the application of biochemical tests, namely, Gram staining and the 3% KOH test, in accordance with established methodologies [10].

### Isolation of bacteria from the rhizospheric population

Bacterial isolation from the rhizosphere was conducted via the serial dilution method. Specifically, 0.1 µL of the solution was extracted from the 5th, 6th, and 7th tubes and subsequently spread evenly on NA media plates via glass beads. These inoculated plates were then incubated at 25 °C for a period of 24 h. Following incubation, the colonies were subjected to purification via the streaking method. Morphological identification of all the purified isolates was performed by assessing characteristics such as color and colony type and conducting biochemical tests, including Gram staining and the 3% KOH test, as outlined by Mubeen et al. [10].

### DNA extraction and PCR

DNA from the bacterial isolate was extracted via a modified CTAB method. The obtained DNA pellets were washed with ethanol and dried for subsequent use in polymerase chain reaction (PCR), following the procedure outlined by Gupta [11]. The amplification of the 16 S rRNA gene was performed via PCR via forward and reverse primers in a reaction mixture containing dNTPs, buffer, 0.5 µM each primer, 2 mM MgCl2, and 2 units of Taq polymerase. The PCR protocol consisted of denaturation at 95 °C, annealing at 60 °C, and extension at 72 °C for 20, 20, and 45 s, respectively, over 34 cycles. A final autolextension step was performed at 72 °C for 5 min. After amplification, the PCR products were stained with ethidium bromide (100 µg/ml) and separated on a 1.0% (w/v) agarose gel in 0.5X TBE buffer. The bands were visualized under an ultraviolet transilluminator. Following gel electrophoresis, the amplified 16 S rRNA gene fragments were purified and sequenced. The resulting sequences were then subjected to BLAST analysis (Basic Local Alignment Search Tool) to compare them against the NCBI GenBank database for species identification. The highest sequence similarity was used to identify the bacterial isolate at the species level [11, 12].

### Extraction and quantification of NAHL

N-acyl homoserine lactones (NAHLs) were extracted from the pathogenic culture and incubated for 16 h by employing an ethyl acetate solution in equal proportions. The obtained supernatant was then added onto thin-layer chromatography (TLC) plates and overlaid with soft agar containing biosensors.

### NAHL production and degradation assay using liquid cultures

The Quencher strain was introduced into NAHLs containing Luria–Bertani medium (LBM) and allowed to incubate for 16 h. A 20 ml aliquot was subsequently withdrawn from the tubes and applied as discrete spots onto TLC plates. These plates were overlaid with soft agar preinoculated with biosensors. The identification of the CV026 strain was established by the manifestation of a violet hue, while the presence of NTLR4 was discerned through the development of blue pigmentation.

### Coculturing

Before the microbial consortia were developed, compatibility studies were conducted to evaluate interactions among selected strains. These tests, including coculture assays and growth rate comparisons, revealed positive interactions that enhanced growth and metabolic activity. This preliminary evaluation is essential for the formation of a robust microbial consortium to improve plant growth and soil health. Cocultivation was executed in a shaking incubator for 16 h at 250 rpm and 28 °C in LB media (LBM). The optical density of the bacterial cultures was standardized to 0.5 at a wavelength of 650 nm via a spectrophotometer. The cultures were subsequently reinoculated or coinoculated onto fresh LB medium. The samples were serially plated onto fresh LBM plates at two-hour intervals. The plated samples were incubated for 16 h, and the resulting colonies were quantified, considering the dilution factor.

### Harvesting of flowers and corms

The initial or second floral buds, poised for imminent blooming, were selectively harvested during both the morning and evening sessions. A precise incision between the stem and leaves was made via a sharp knife. The retrieval of corms involves excavation from the soil, yielding one or two substantial corms accompanied by 20–30 cormels. The harvested cormels were subsequently utilized for tissue culture applications.

### Statistical analysis

The data were analyzed via IBM SPSS V.22. One-way analysis of variance (ANOVA) was performed to compare the effects of various treatments. Fisher’s complete randomized block design (RCBD) was used for the experimental setup. Significant differences among the treatment means were determined via post hoc Tukey’s HSD test, with a significance threshold set at *p* < 0.05. Additionally, Pearson’s correlation method [12] was used to analyze the relationships between the variables.

## Results

### Disease assessment of gladiolus corms sown under natural conditions

Major gladiolus diseases, specifically gladiolus leaf spot, fusarium rot of gladiolus (corm rot), and gladiolus bacterial scab (corm scab), were identified as prevalent in the experimental field. A visual assessment revealed that approximately 15–20 plants presented signs of bacterial scab infection, 10 plants were affected by corm rot, and approximately 5–7 plants presented leaf spot. Subsequent isolation and identification of the pathogens confirmed a disease incidence of 10% for corm scab, 7% for corm rot, and 3% for leaf spot.

### Isolation and identification of associated pathogens

**Fig. 1:**
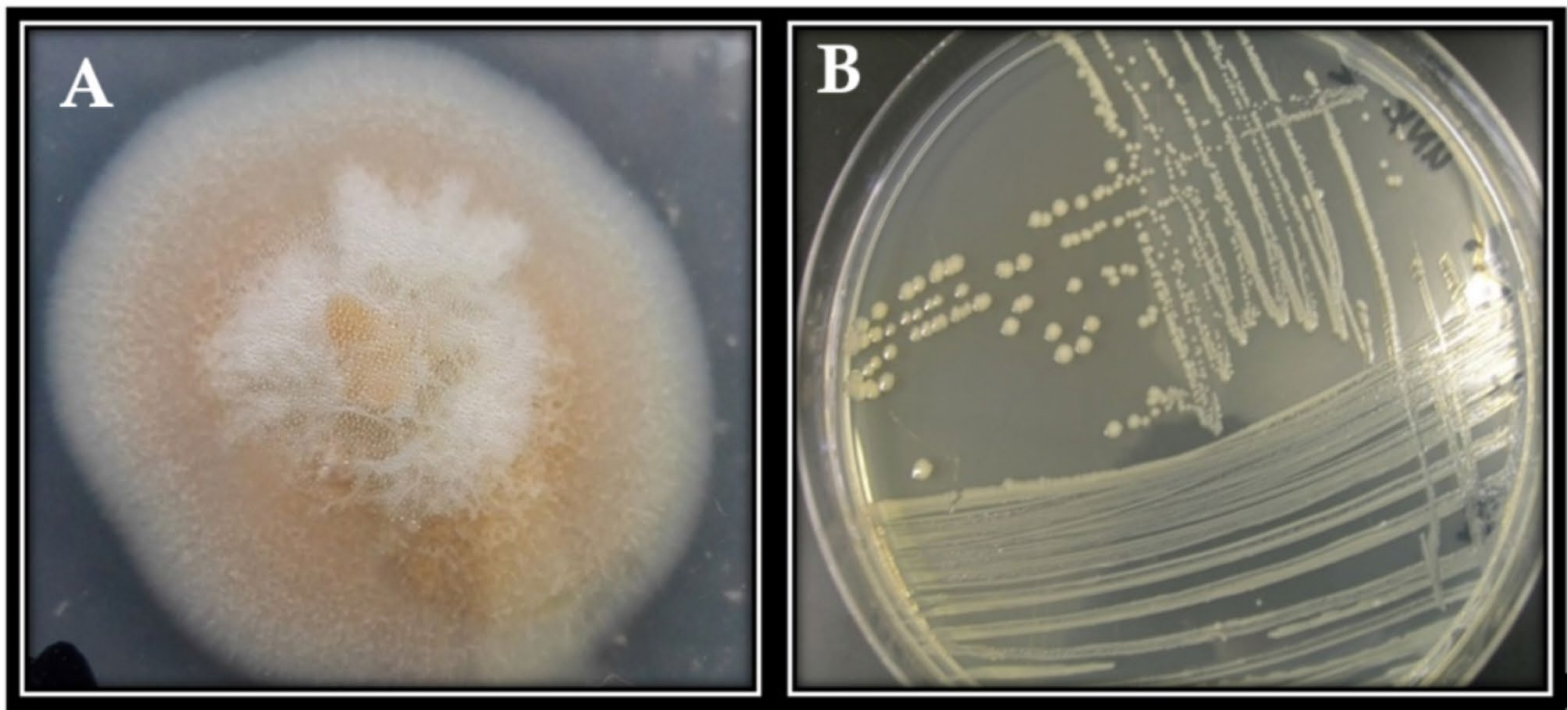
**A**, fungal; and **B**, bacterial isolates, their colony color and shape

In the process of isolating bacterial pathogens, nutrient agar media was utilized under aseptic conditions. The cultivation of infected corms and plant parts on the specified media facilitated the isolation of both fungal and bacterial pathogens (Fig. 1).

### Soil sample collection and colonies of bacterial isolates from serial dilutions

**Fig. 2:**
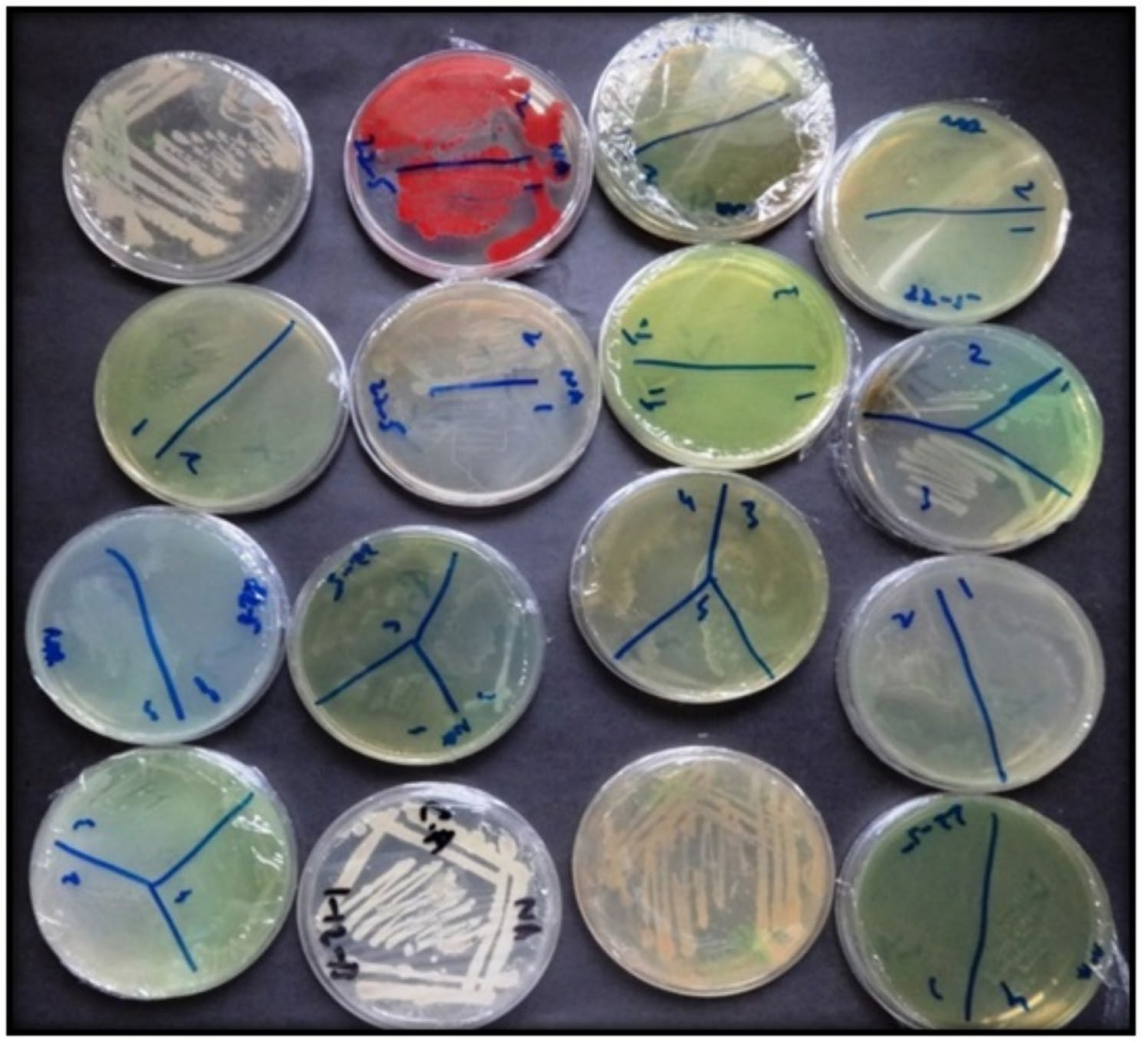
Colonies of rhizospheric bacterial isolates

Rhizospheric soil samples were obtained from both healthy plants and treated plants to ensure comprehensive analysis of microbial dynamics. Soil samples were carefully taken from the root zone at a depth of approximately 5–10 cm via a sterile trowel. The collected samples were placed in sterile polyethylene bags and transported to the laboratory on ice for further analysis. For each plant, three replicate samples were collected to account for variability. These samples were processed immediately or stored at 4 °C for no longer than 24 h before analysis. Sixteen bacterial isolates were isolated from the soil samples via a serial dilution process and were purified on the basis of the color, shape and morphology of the colonies (Fig. 2).

### N-Acyel homoserine lactone identification

**Fig. 3:**
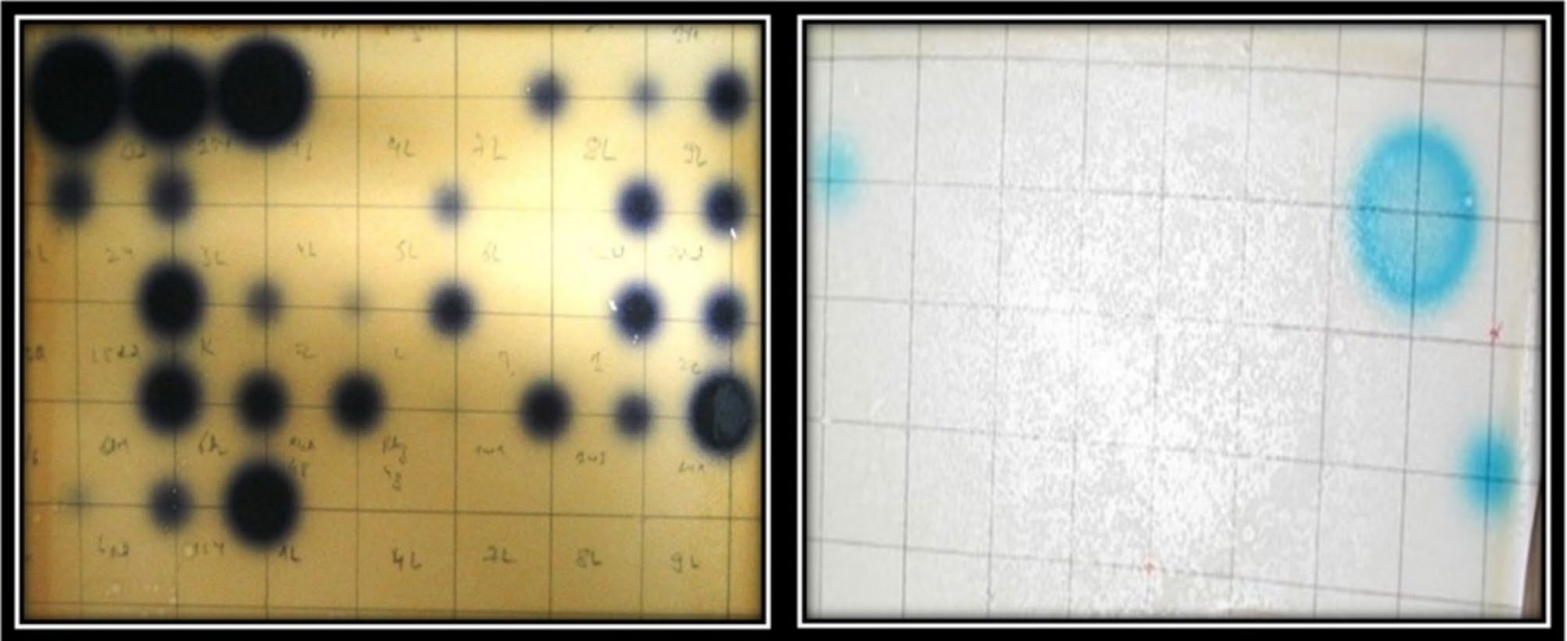
Violet color produced from CV026 and blue color produced from NTLR4

Five strains demonstrated degradative properties, prompting a more in-depth examination to assess their potential as quorum sensing (QS) inhibitors. Additionally, three strains exhibited N-acyl homoserine lactone (NAHL) production properties, categorizing them as QS producers (Fig. 3).

### N-Acyel homoserine lactone degrader bacteria

Bacterial isolates were identified through BLAST searches of PCR-amplified 16 S rRNA gene sequences. Five of them exhibited NAHL degrader activities, as shown in Table 2, and N-Acyel homoserine lactone-producing bacteria, as shown in Table 3.

**Table 2:** NAHL degraders

Strain	Closest Relative	NAHL Degradation C6 Oxo C8, C10		QQ
Q1	*Acinobacter sp*. R-26,465	+	+	+
Q2	*Bacillus cereus* H316S	+	+	+
Q3	*Bacillus subtilis*. isolate VIT SPS2	+	+	+
Q4	*Enterobacter cloacoe*	+	+	+
Q5	*Bacillus subtilis isolate HPCAQA6*	+	+	+

**Table 3:** N-Acyel homoserine lactone producer Bacteria

Strain	Closest Relative	NAHL Producer C6 Oxo C8, C10		QS
Q1	*Ochrobactrum sp*. R-26,465	+		+
Q2	*Pseudomonas fluorescens*	+	+	+
Q3	*Burkholderia* sp. isolate N3P2	+	+	+

#### Coculturing

**Fig. 4:**
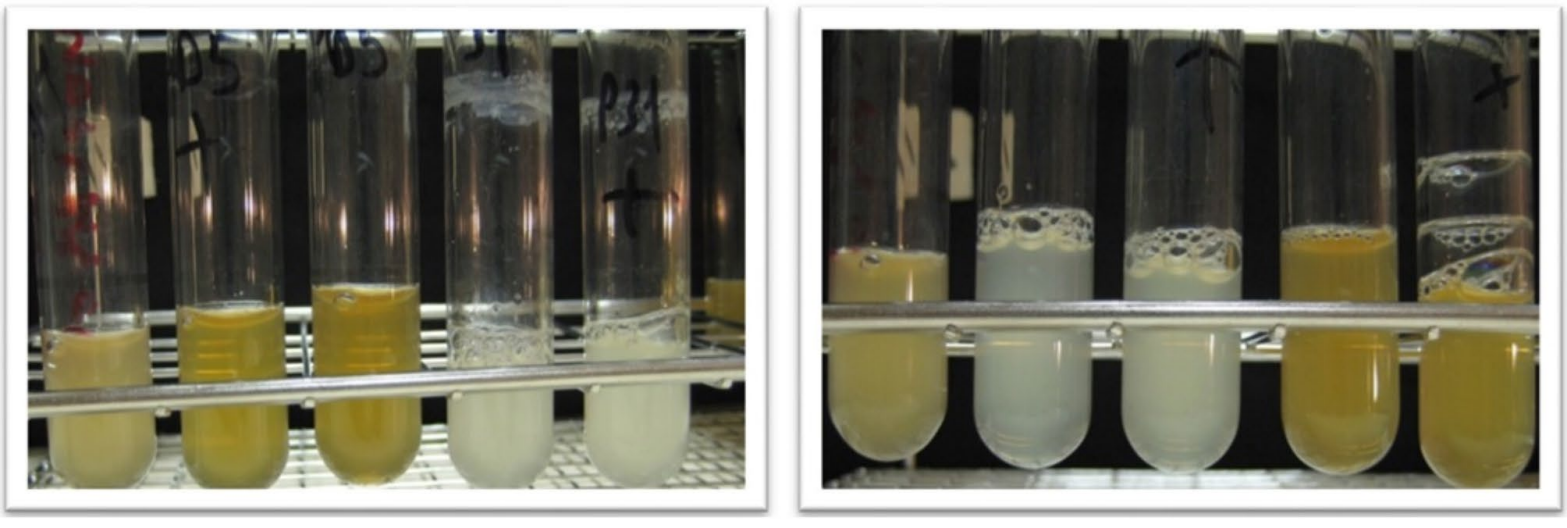
Co-culture of different rhizospheric bacterial isolates

One strain exhibited a suppressive effect on the growth of *B. gladioloi*, whereas another strain was inhibited when cultured in Luria Bertani medium (LBm). Those strains that neither influenced nor were influenced were selected for subsequent pathogenicity quenching assays (Fig. 4).

#### Molecular characterization of QS and QQ

PCR amplification was conducted with universal primers to amplify DNA sequences. The obtained DNA sequences were subsequently compared to those available in the National Center for Biotechnology Information (NCBI) database to ascertain the bacterial species. Through a comparative analysis of the 16 S rRNA sequences of the clones with the reference species archived in GenBank, it was deduced that the clones were associated with specific species, including *Bacillus cereus*,*Bacillus subtilis*,*Actinobacteria*,*Enterobacter*,*Ochrobactrum* sp., *Pseudomonas*, and *Burkholderia.*

#### Symptoms observed

**Fig. 5:**
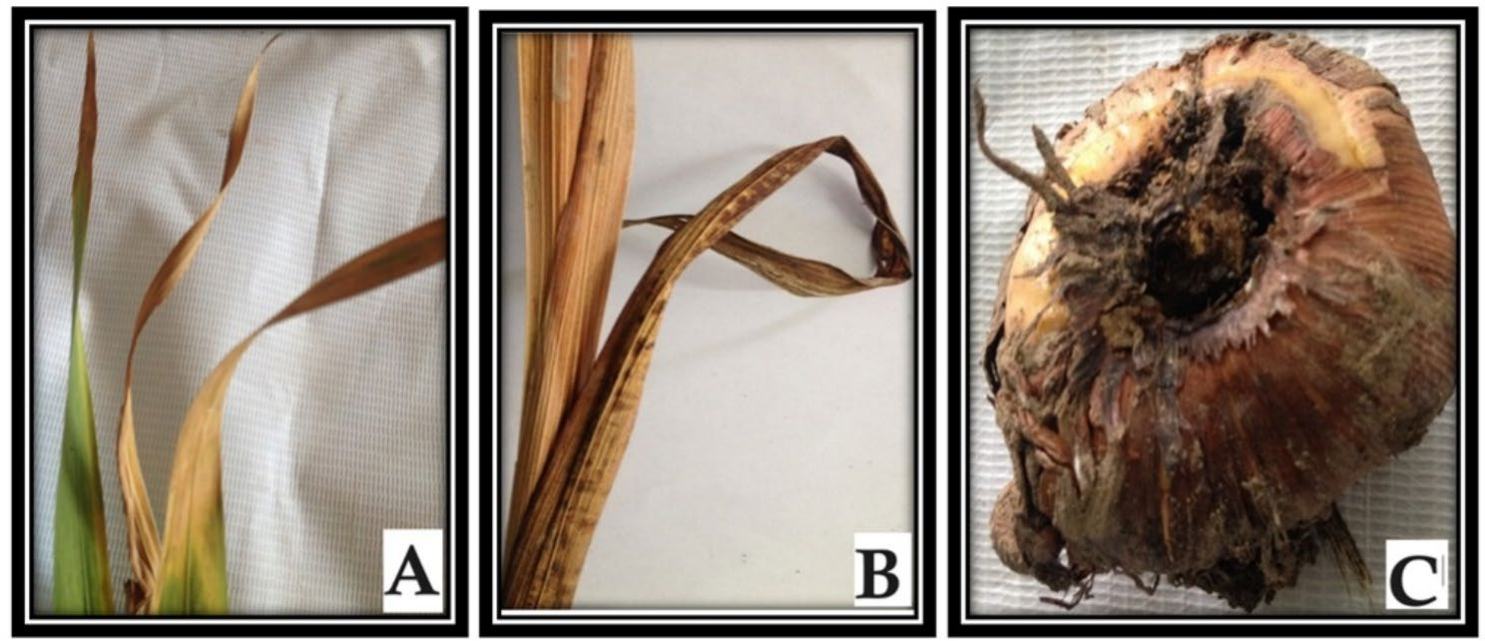
(**A**) Yellowing of leaves. (**B**) Death of plants. (**C**) Rotted corm

Symptoms were induced in the control block, manifesting as early-stage yellow and necrotic spots. The progression of the disease resulted in leaf yellowing, leading to tissue necrosis, as illustrated in Fig. 5(A). Under severe disease conditions, as depicted in Fig. 5(B), plant mortality ensues, accompanied by observable streaking on the leaves of certain plants. Furthermore, the corms completely rotted, as shown in Fig. 5(C), and their size was notably reduced compared with that of healthy corms.

#### Impact of microbial consortia on plants

The predominant portion of the plant population, accounting for 95% of the total population, displayed robust health. Conversely, all plants in the control group succumbed to infection by *B. gladioli*. The application of irrigation to a consortium of microbial entities has shown the capacity to suppress the activities and growth of quorum sensing (QS) bacteria, thereby sustaining plant health. In contrast, the control experiment, which lacked irrigation, led to complete disease incidence and yield losses of 100%. The quencher consortium not only alleviated the impact of the disease on gladiolus but also contributed to increased growth.

#### Effects of different treatments on germination percentage

**Fig. 6:**
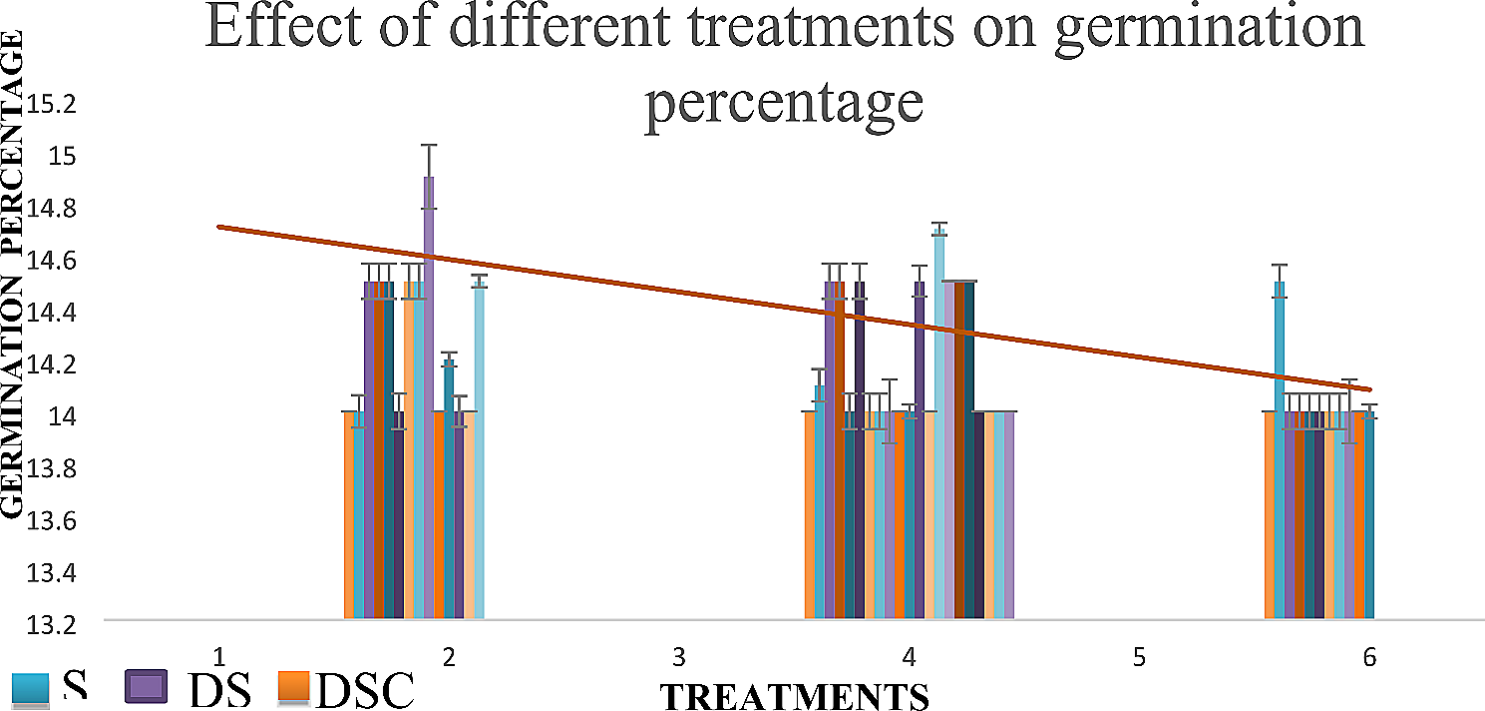
Germination percentage of treatments in which DS resulted in more germination than did S or DSC

Three distinct treatments were implemented, namely, scaled, descaled, and descaled and cut in half. These treatments resulted in minimal disparities in germination percentages, with 14.9% descaled, 14.7% scaled, and 14.5% descaled and cut in half. The graphical representation in Fig. 6 delineates the nuanced differences in percentages.

The germination pattern presented an intriguing aspect, with marginal variations observed between the germination trends of the scaled (S) treatment compared with those of the descaled (DS) and descaled and cut in half (DSC) treatments. The preference among farmers for greater diversity in germination arises from the broader spectrum of biological factors over time, facilitating a more comprehensive observation of local market trends. Consequently, the descaled and cut in half (DSC) method of sowing is recommended for farmers.

#### Effects of different treatments on plant growth

**Fig. 7:**
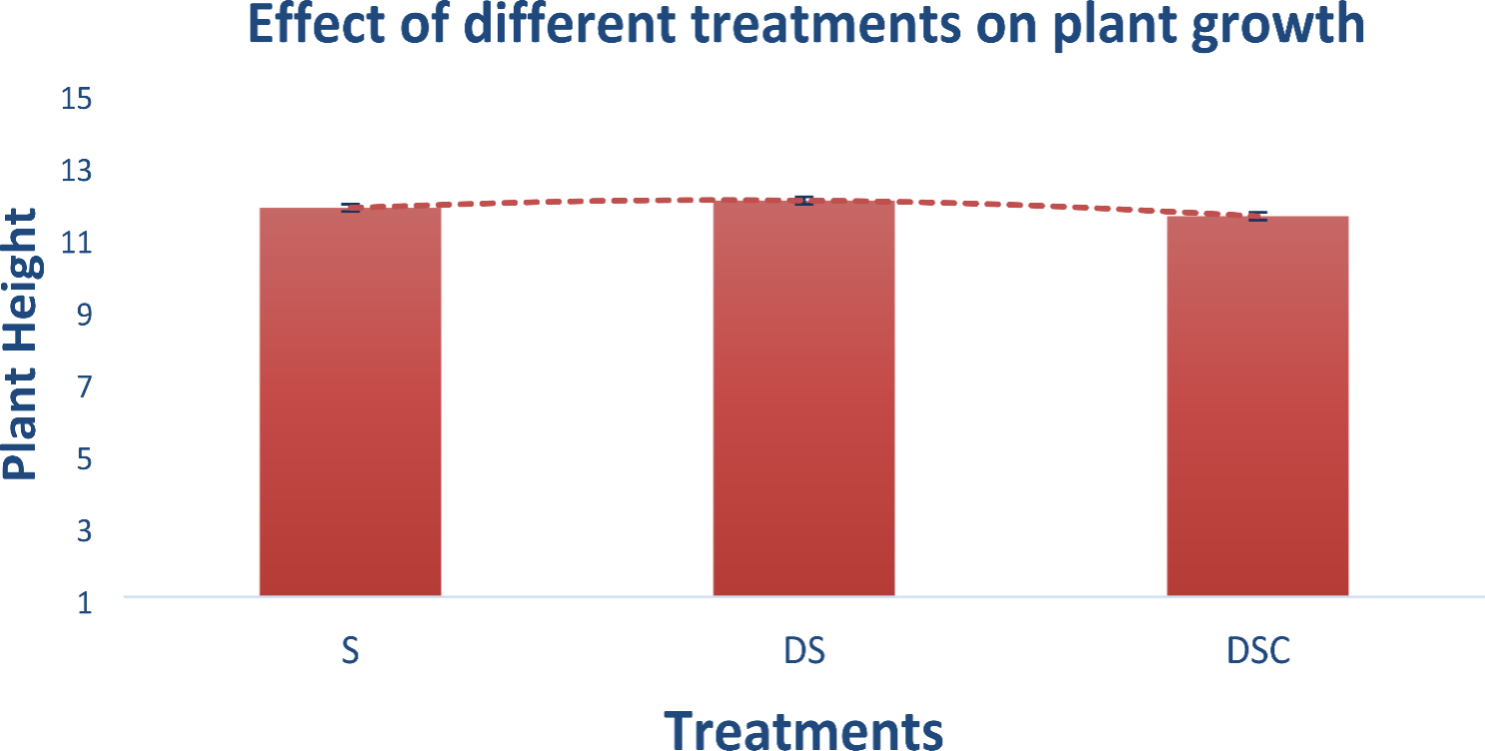
DS results in more (0.25%) plant growth than the other two treatments do

There was no significant difference in terms of plant growth among the treatments. Compared with S and DSC, DS resulted in 0.25% greater plant growth. The polynomial trend line shows that DS resulted in more plant growth, as shown in Fig. 7.

#### Effect of treatments on flower yield

**Fig. 8:**
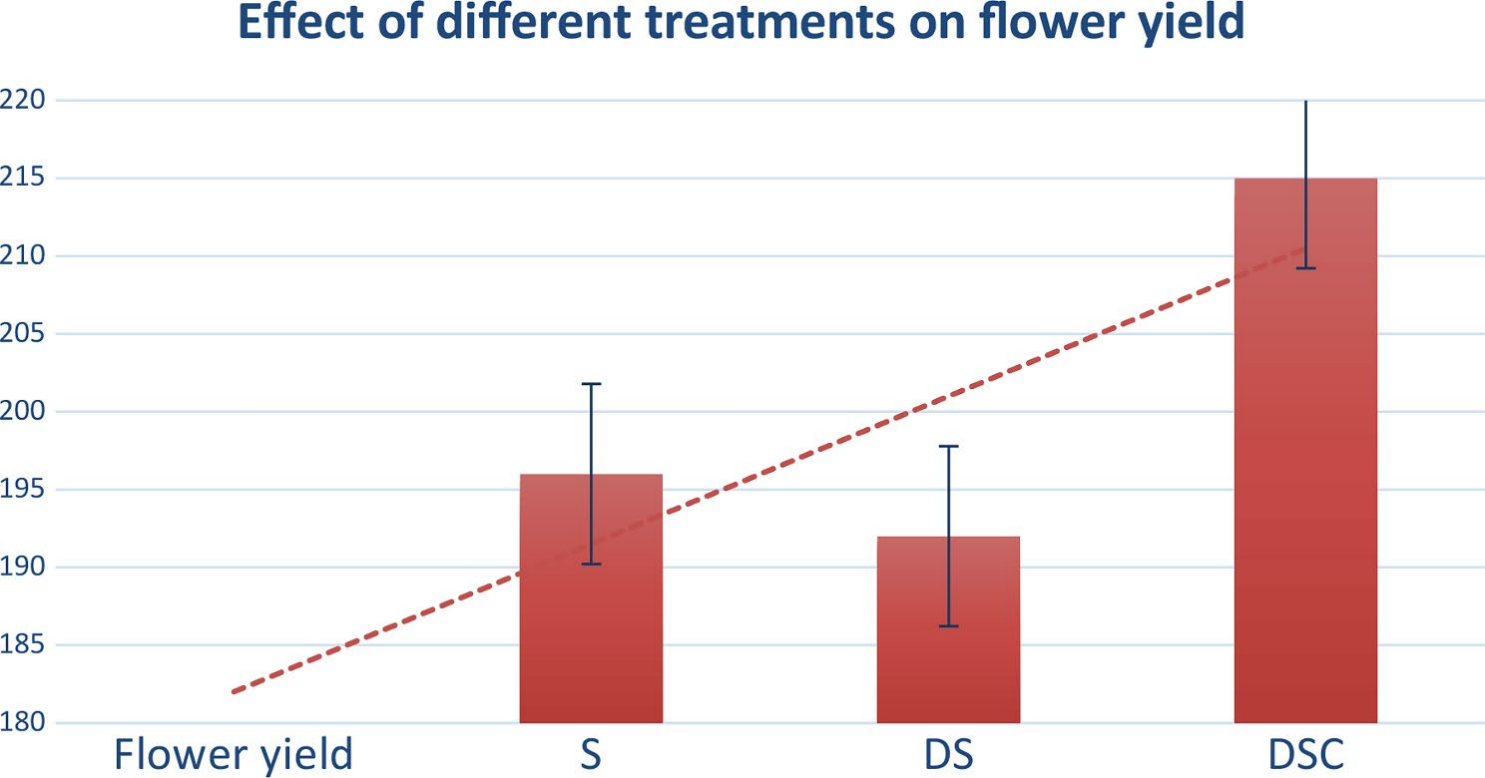
DSC results in the maximum flower yield, followed by S and DS

The scale and cut halves yielded more flowers than the scale and scale halves did (Fig. 8).

#### Percentage corm yield

**Fig. 9:**
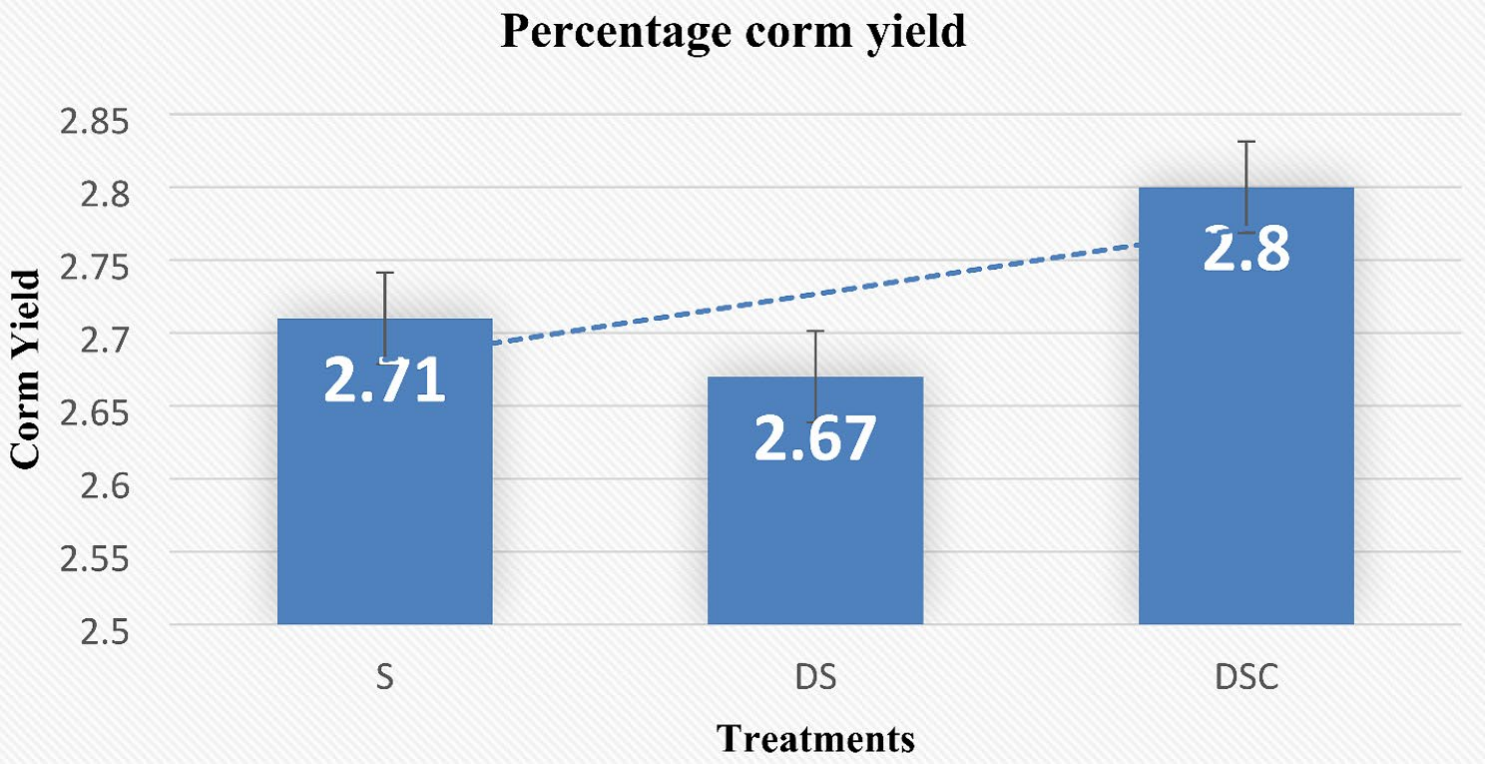
DSC results in a greater corm yield

DSC produced more corms than did S and DS. In terms of yield percentage, the DSC was 2.8%, whereas S was 2.71%, and DS was 2.67% yield (Fig. 9).

#### Trend of corm yield

**Fig. 10:**
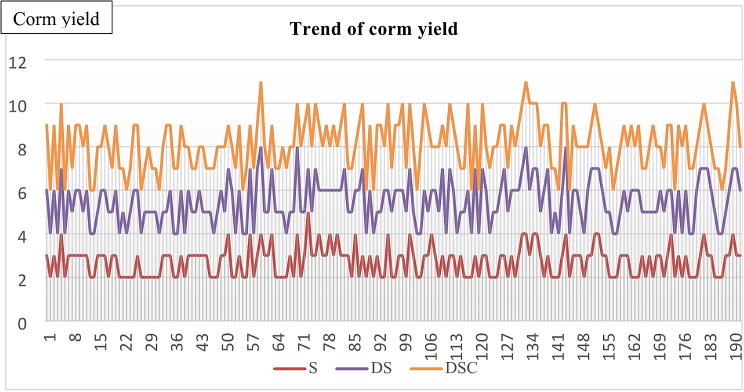
DSC shows more variation in the yield of the corms

Scale has less yield variance than does descale and cut. Figure 10 shows the corm yield trend.

#### Trend of germination percentage

**Fig. 11:**
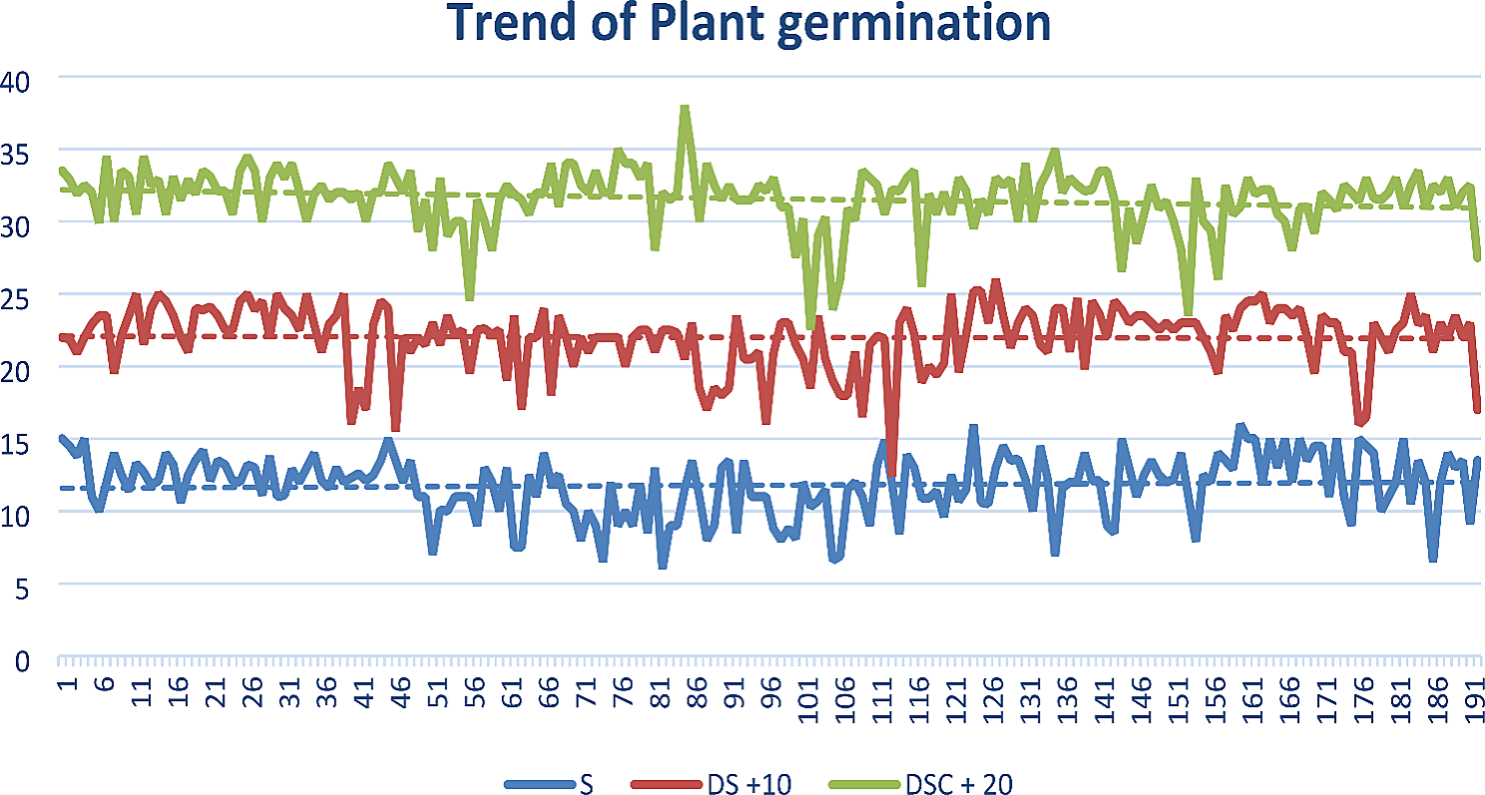
DSC shows more variation in terms of the percentage of plant germination

The pattern of germination was especially interesting, with the S treatment resulting in little change from DS to DSC, as demonstrated in Fig. 11.

**Fig. 12:**
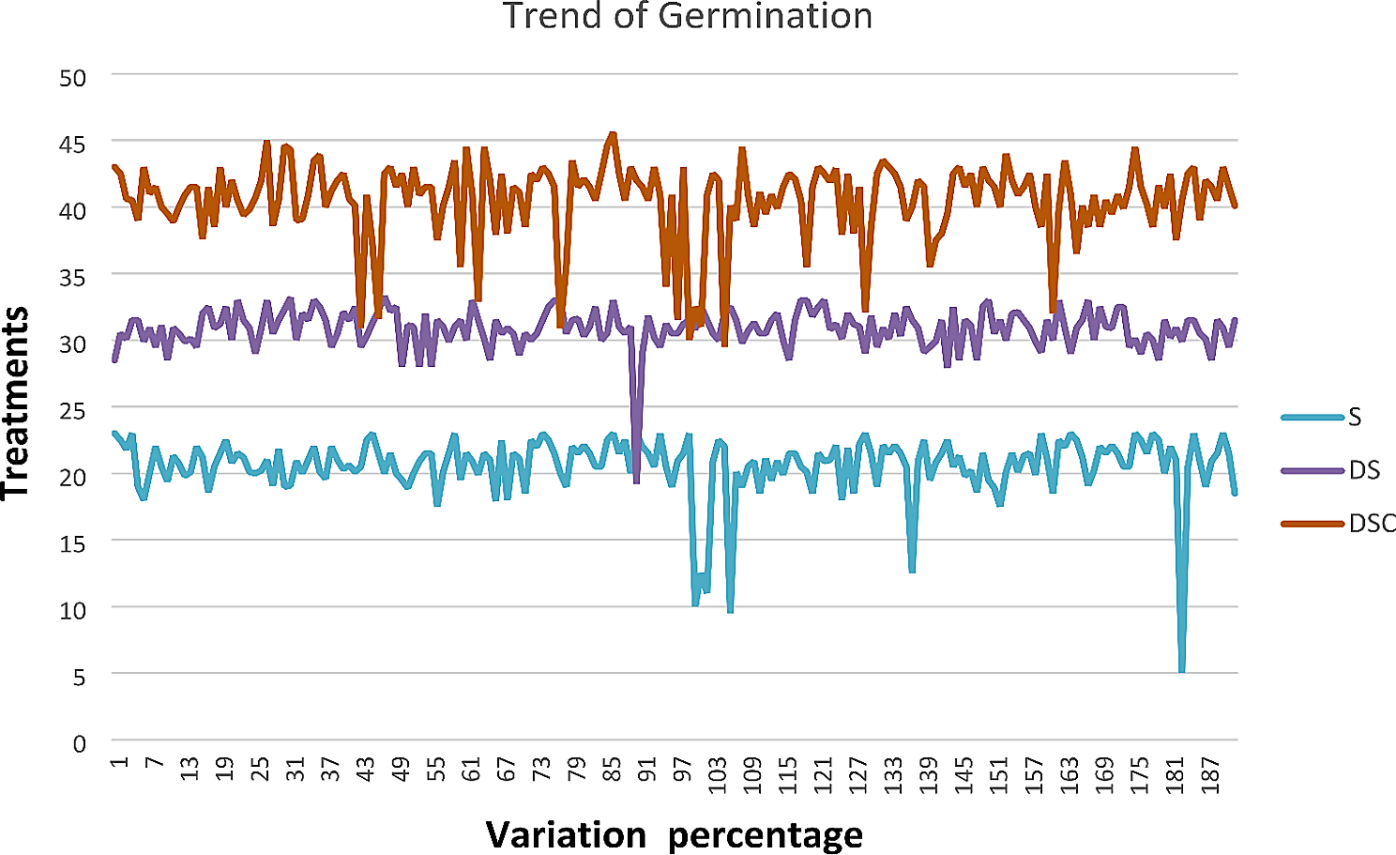
Trend of Germination

More variation was shown in the DSC treatment than in the S and DS treatments, and the results show that DSC could be used for sowing purposes, as shown in Fig. 12.

## Discussion

Gladiolus holds the second most prominent position in the cut flower industry, constituting 60% of the $11 million in the global flower trade, and is anticipated to triple by 2025 [13]. However, various bacterial and fungal diseases pose substantial threats to gladiolus production, with *B. gladioli* pv. *gladioli*, a bacterial pathogen, is associated with significant disease incidence, necessitating effective control measures. The interplay among bacterial species within the rhizosphere introduces competition for the host plant, providing protection against other diseases and increasing pathogenicity. Plant-associated strains containing AHLases or amidases can inhibit the pathogenic activities of other plant diseases, as exemplified by species such as *B. thuringiensis*, *P. fluorescens*, and *Arthrobacter* spp [9]. The results of this study support those of previous studies that explored the effectiveness of the use of QSIs and QQ bacteria in agricultural contexts. The isolation and utilization of bacterial strains that centered QS signals and demonstrated QQ activity from the gladiolus rhizosphere produced certain key findings that corroborated the findings of previous studies [9, 14, 15].

First, the successful isolation of diverse bacterial species, including *Bacillus cereus*, *B. subtilis*,*Actinobacteria*,*Enterobacter*,*Ochrobactrum* sp., *Pseudomonas*, and *Burkholderia*, is consistent with prior studies reporting the presence of these bacteria in various rhizospheres and their potential roles in plant health and disease suppression [16]. These bacteria are primarily involved in stimulating plant growth and nitrogen fixation and protecting against diseases through the formation of secondary metabolites and enzymes that breakdown signals [17].

Similarly, *Chromobacterium violaceum* and *Agrobacterium tumefaciens* also exhibited QSI properties, implying that some bacterial strains inhibit the ability of the pathogen to prevent disease transmission [9]. The identified strains belonging to *Acinobacter* sp., *Bacillus cereus*,*Bacillus subtilis*, and *Enterobacter cloacoe*, were found to have effective QS inhibition similar to other reports, indicating that the genera have enzymes that are able to degrade N-acyl homoserine lactones (NAHLs), which are involved in QS [18].

The practical application of quorum-quenched biocontrol agents revealed a high level of disease control and improved plant health, indicating a lower number of plants infected by pathogens in the quorum-quenched consortium-treated plants than in the untreated plants. This method was previously proposed as a way to interfere with the QS systems of pathogens and decrease their toxicity [14]. The findings of this study raise the possibility that QQ bacteria shield crops from diseases and enhance their health, making these findings meaningful for agriculture.

The differences in germination percentage and plant growth depending on the method of seeding indicate that proper planting techniques should be employed to achieve the maximum yield possible. The DSC method, which yielded the highest corm yield as well as flower production, suggested that some sowing techniques increase the impact of QQ bacteria. In this context, the present findings support the results obtained by Hariprasad and Niranjana [15], who revealed that selected agronomic management practices improve microbial inoculants to increase plant health.

## Conclusion

This study’s findings corroborate the potential of using QS inhibitors and QQ bacteria as a viable strategy for disease management in crops. The isolation of effective bacterial strains and their successful field application underscore the importance of integrating microbiological approaches with conventional agricultural practices. Future research should focus on long-term field trials, explore the interactions between QQ bacteria and different crops, and assess the economic viability of these strategies for large-scale agricultural adoption.

## Data Availability

The datasets used and/or analyzed during the current study available from the corresponding authors on reasonable request.

## Abbreviations

QS: Quorum Sensing
QQ: Quorum Quenching
NA: Nutrient Agar
PCR: Polymerase Chain Reaction
DSC: Descale and Cut Half
